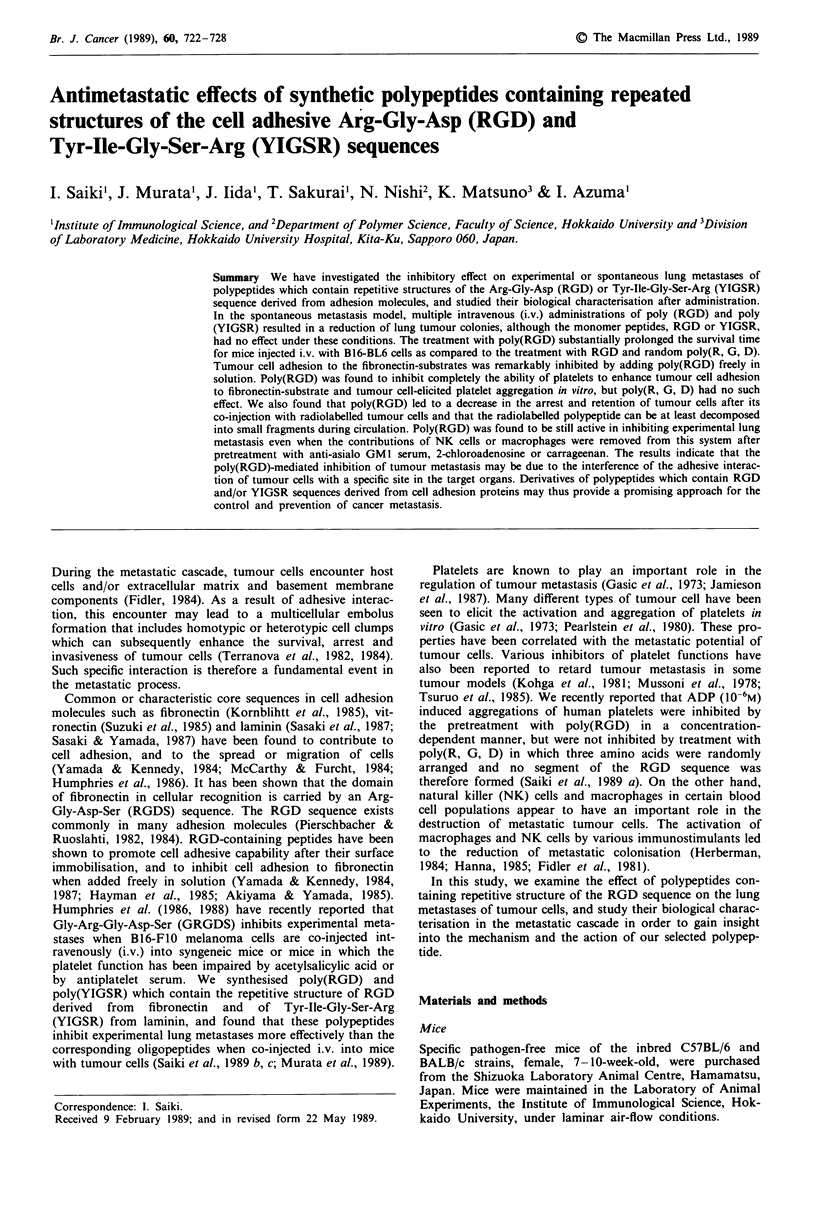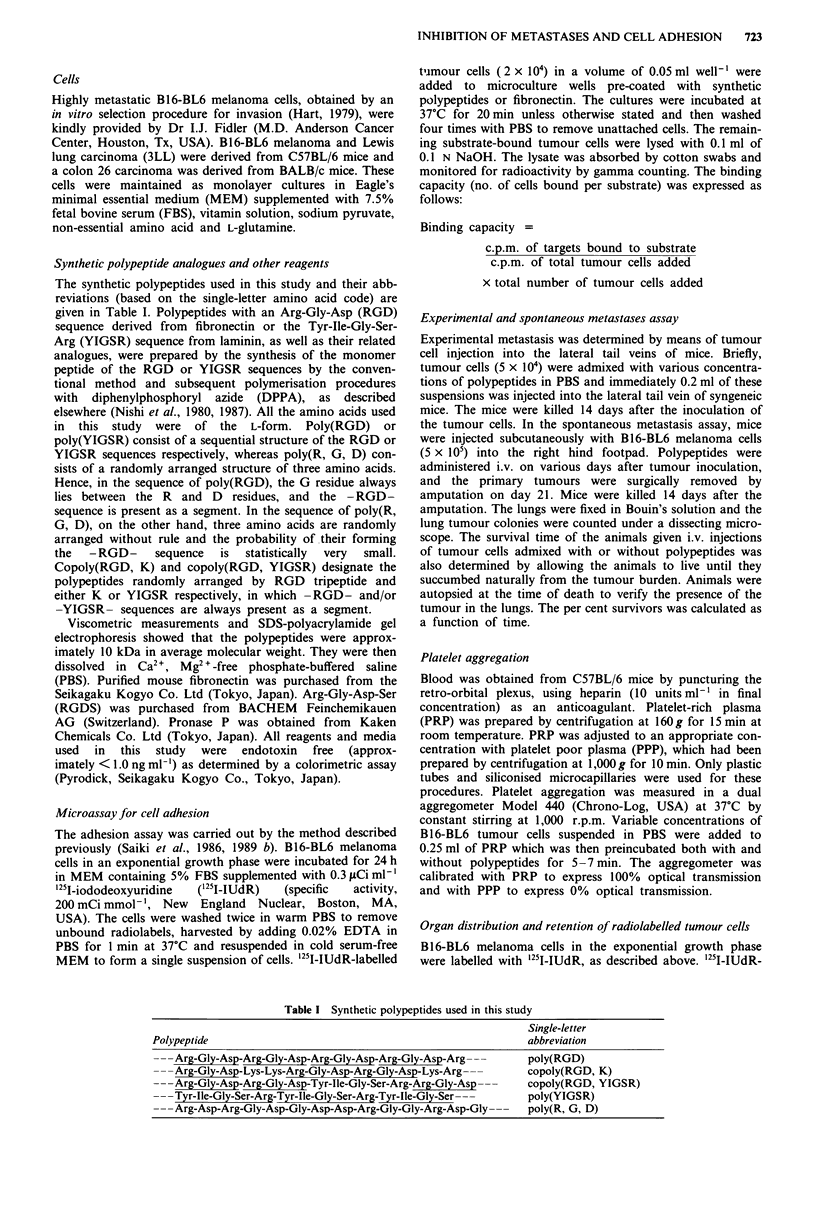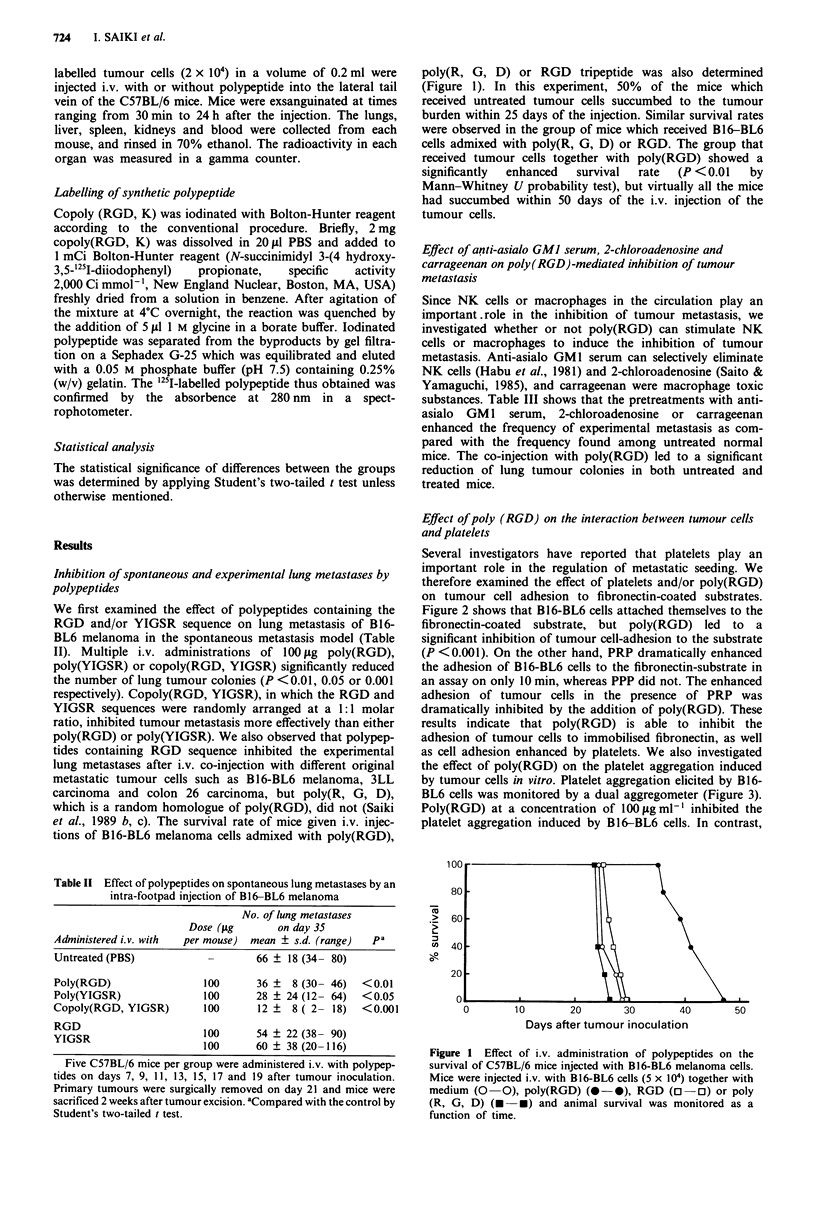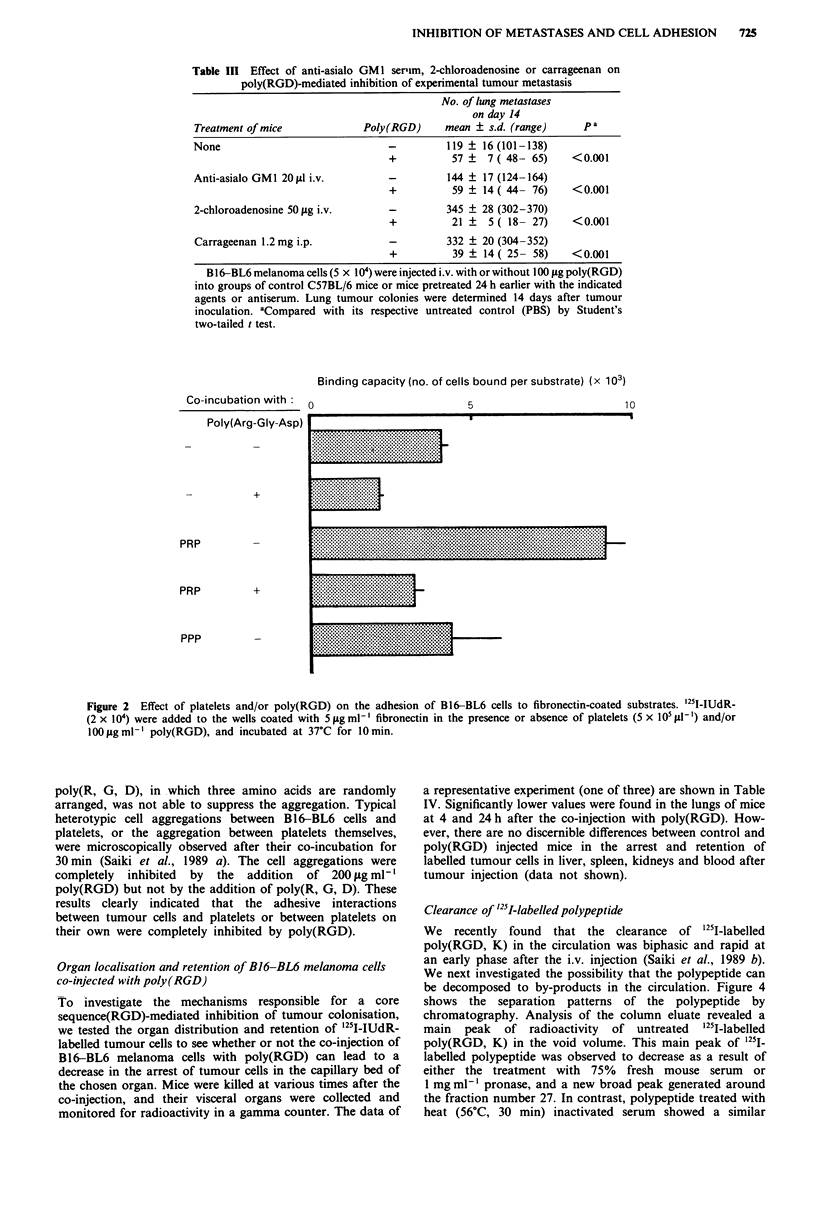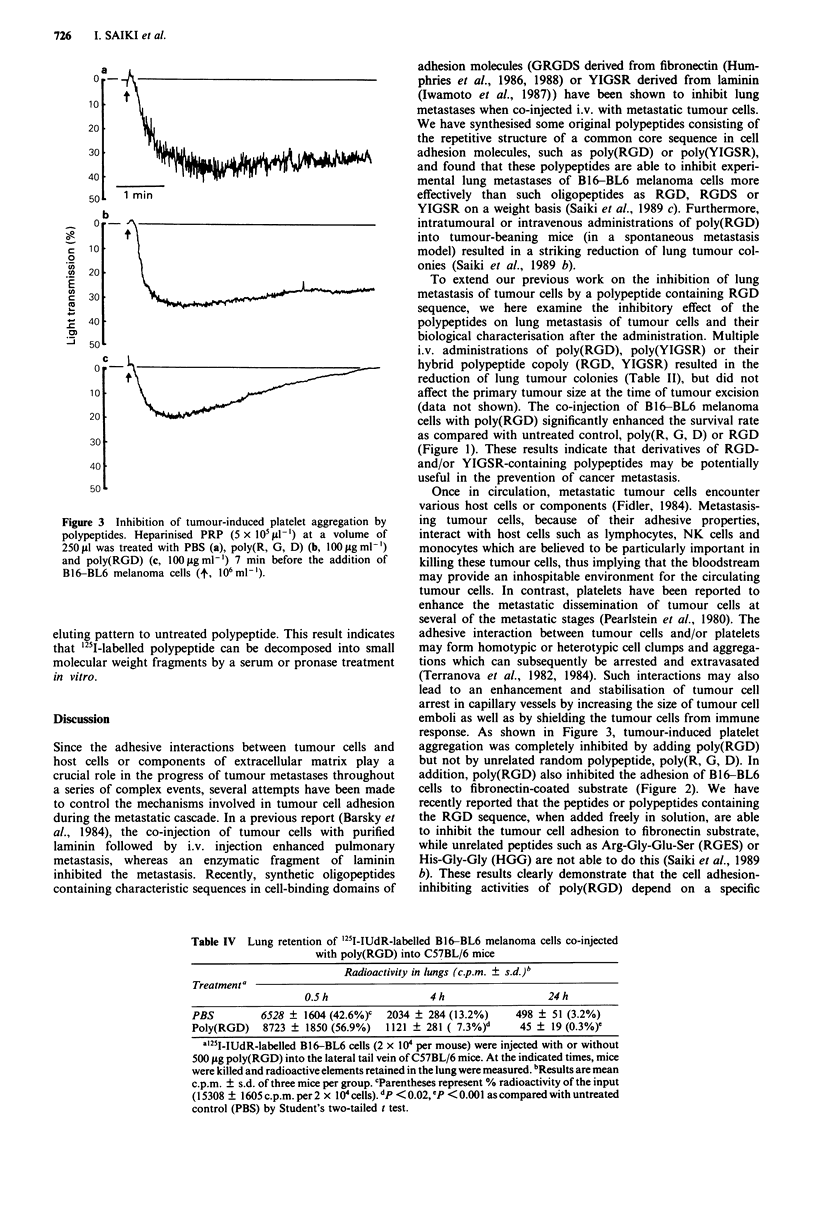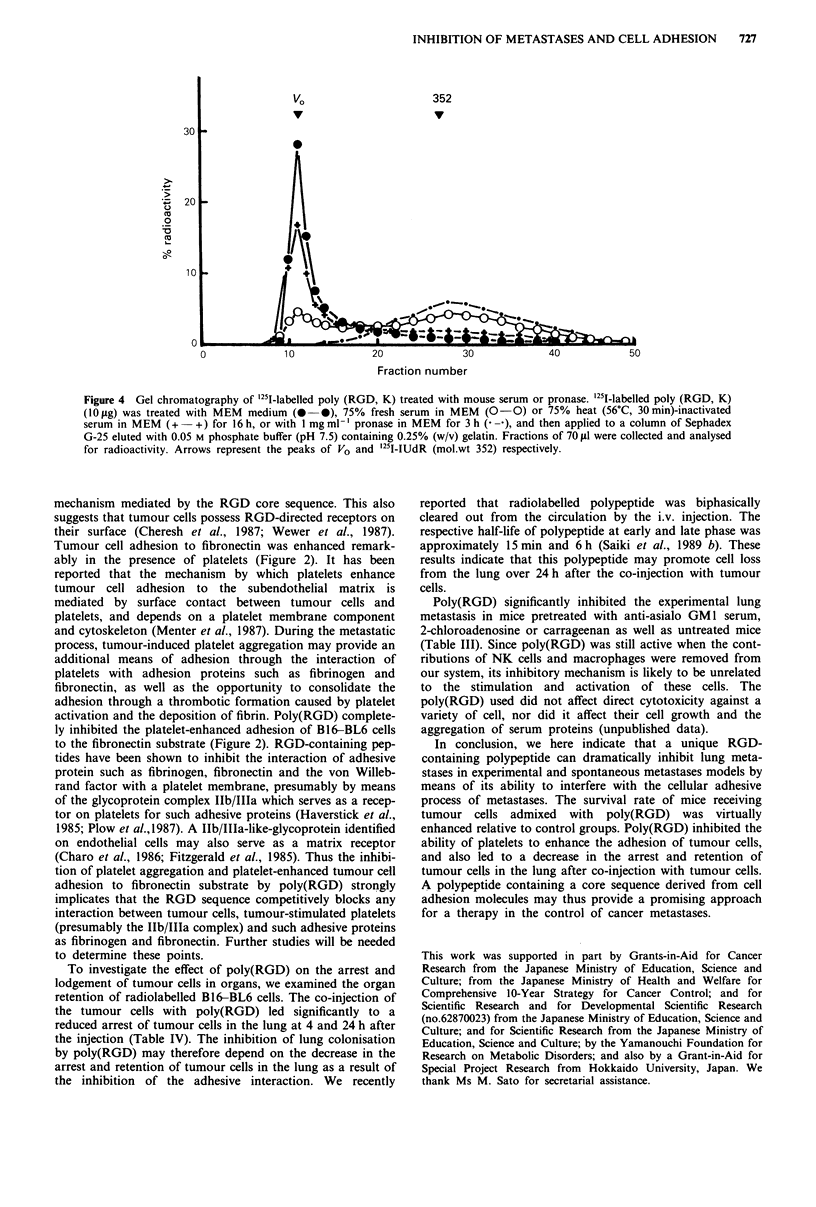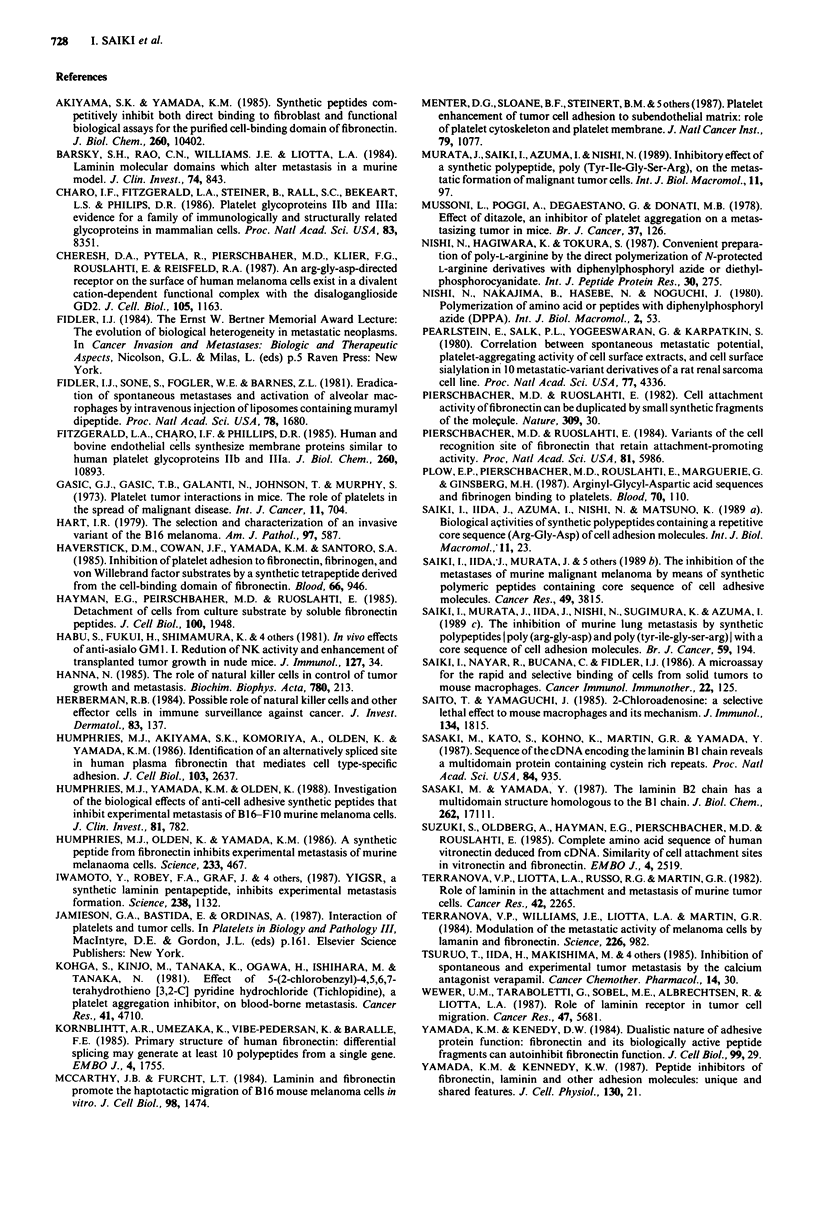# Antimetastatic effects of synthetic polypeptides containing repeated structures of the cell adhesive Arg-Gly-Asp (RGD) and Tyr-Ile-Gly-Ser-Arg (YIGSR) sequences.

**DOI:** 10.1038/bjc.1989.347

**Published:** 1989-11

**Authors:** I. Saiki, J. Murata, J. Iida, T. Sakurai, N. Nishi, K. Matsuno, I. Azuma

**Affiliations:** Institute of Immunological Science, Faculty of Science, Hokkaido University, Sapporo, Japan.

## Abstract

We have investigated the inhibitory effect on experimental or spontaneous lung metastases of polypeptides which contain repetitive structures of the Arg-Gly-Asp (RGD) or Tyr-Ile-Gly-Ser-Arg (YIGSR) sequence derived from adhesion molecules, and studied their biological characterisation after administration. In the spontaneous metastasis model, multiple intravenous (i.v.) administrations of poly (RGD) and poly (YIGSR) resulted in a reduction of lung tumour colonies, although the monomer peptides, RGD or YIGSR, had no effect under these conditions. The treatment with poly(RGD) substantially prolonged the survival time for mice injected i.v. with B16-BL6 cells as compared to the treatment with RGD and random poly(R, G, D). Tumour cell adhesion to the fibronectin-substrates was remarkably inhibited by adding poly(RGD) freely in solution. Poly(RGD) was found to inhibit completely the ability of platelets to enhance tumour cell adhesion to fibronectin-substrate and tumour cell-elicited platelet aggregation in vitro, but poly(R, G, D) had no such effect. We also found that poly(RGD) led to a decrease in the arrest and retention of tumour cells after its co-injection with radiolabelled tumour cells and that the radiolabelled polypeptide can be at least decomposed into small fragments during circulation. Poly(RGD) was found to be still active in inhibiting experimental lung metastasis even when the contributions of NK cells or macrophages were removed from this system after pretreatment with anti-asialo GM1 serum, 2-chloroadenosine or carrageenan. The results indicate that the poly(RGD)-mediated inhibition of tumour metastasis may be due to the interference of the adhesive interaction of tumour cells with a specific site in the target organs. Derivatives of polypeptides which contain RGD and/or YIGSR sequences derived from cell adhesion proteins may thus provide a promising approach for the control and prevention of cancer metastasis.


*Br. J. Cancer* (1989), **60**, 722–728 © The Macmillan Press Ltd., 1989

### Antimetastatic effects of synthetic polypeptides containing repeated structures of the cell adhesive Arg-Gly-Asp (RGD) and Tyr-Ile-Gly-Ser-Arg (YIGSR) sequences

I. Saiki[1], J. Murata[1], J. Iida[1], T. Sakurai[1], N. Nishi[2], K. Matsuno[3] & I. Azuma[1]

[1]*Institute of Immunological Science, and* [2]*Department of Polymer Science, Faculty of Science, Hokkaido University and* [3]*Division of Laboratory Medicine, Hokkaido University Hospital, Kita-Ku, Sapporo 060, Japan.*

**Summary** We have investigated the inhibitory effect on experimental or spontaneous lung metastases of polypeptides which contain repetitive structures of the Arg-Gly-Asp (RGD) or Tyr-Ile-Gly-Ser-Arg (YIGSR) sequence derived from adhesion molecules, and studied their biological characterisation after administration. In the spontaneous metastasis model, multiple intravenous (i.v.) administrations of poly (RGD) and poly (YIGSR) resulted in a reduction of lung tumour colonies, although the monomer peptides, RGD or YIGSR, had no effect under these conditions. The treatment with poly(RGD) substantially prolonged the survival time for mice injected i.v. with B16-BL6 cells as compared to the treatment with RGD and random poly(R, G, D). Tumour cell adhesion to the fibronectin-substrates was remarkably inhibited by adding poly(RGD) freely in solution. Poly(RGD) was found to inhibit completely the ability of platelets to enhance tumour cell adhesion to fibronectin-substrate and tumour cell-elicited platelet aggregation *in vitro*, but poly(R, G, D) had no such effect. We also found that poly(RGD) led to a decrease in the arrest and retention of tumour cells after its co-injection with radiolabelled tumour cells and that the radiolabelled polypeptide can be at least decomposed into small fragments during circulation. Poly(RGD) was found to be still active in inhibiting experimental lung metastasis even when the contributions of NK cells or macrophages were removed from this system after pretreatment with anti-asialo GM1 serum, 2-chloroadenosine or carrageenan. The results indicate that the poly(RGD)-mediated inhibition of tumour metastasis may be due to the interference of the adhesive interaction of tumour cells with a specific site in the target organs. Derivatives of polypeptides which contain RGD and/or YIGSR sequences derived from cell adhesion proteins may thus provide a promising approach for the control and prevention of cancer metastasis.

During the metastatic cascade, tumour cells encounter host cells and/or extracellular matrix and basement membrane components (Fidler, 1984). As a result of adhesive interaction, this encounter may lead to a multicellular embolus formation that includes homotypic or heterotypic cell clumps which can subsequently enhance the survival, arrest and invasiveness of tumour cells (Terranova *et al.*, 1982, 1984). Such specific interaction is therefore a fundamental event in the metastatic process.

Common or characteristic core sequences in cell adhesion molecules such as fibronectin (Kornblihtt *et al.*, 1985), vitronectin (Suzuki *et al.*, 1985) and laminin (Sasaki *et al.*, 1987; Sasaki & Yamada, 1987) have been found to contribute to cell adhesion, and to the spread or migration of cells (Yamada & Kennedy, 1984; McCarthy & Furcht, 1984; Humphries *et al.*, 1986). It has been shown that the domain of fibronectin in cellular recognition is carried by an Arg-Gly-Asp-Ser (RGDS) sequence. The RGD sequence exists commonly in many adhesion molecules (Pierschbacher & Ruoslahti, 1982, 1984). RGD-containing peptides have been shown to promote cell adhesive capability after their surface immobilisation, and to inhibit cell adhesion to fibronectin when added freely in solution (Yamada & Kennedy, 1984, 1987; Hayman *et al.*, 1985; Akiyama & Yamada, 1985). Humphries *et al.* (1986, 1988) have recently reported that Gly-Arg-Gly-Asp-Ser (GRGDS) inhibits experimental metastases when B16-F10 melanoma cells are co-injected intravenously (i.v.) into syngeneic mice or mice in which the platelet function has been impaired by acetylsalicylic acid or by antiplatelet serum. We synthesised poly(RGD) and poly(YIGSR) which contain the repetitive structure of RGD derived from fibronectin and of Tyr-Ile-Gly-Ser-Arg (YIGSR) from laminin, and found that these polypeptides inhibit experimental lung metastases more effectively than the corresponding oligopeptides when co-injected i.v. into mice with tumour cells (Saiki *et al.*, 1989 *b*, *c*; Murata *et al.*, 1989).

Correspondence: I. Saiki.
Received 9 February 1989; and in revised form 22 May 1989.

Platelets are known to play an important role in the regulation of tumour metastasis (Gasic *et al.*, 1973; Jamieson *et al.*, 1987). Many different types of tumour cell have been seen to elicit the activation and aggregation of platelets *in vitro* (Gasic *et al.*, 1973; Pearlstein *et al.*, 1980). These properties have been correlated with the metastatic potential of tumour cells. Various inhibitors of platelet functions have also been reported to retard tumour metastasis in some tumour models (Kohga *et al.*, 1981; Mussoni *et al.*, 1978; Tsuruo *et al.*, 1985). We recently reported that ADP ($10^{-6}$M) induced aggregations of human platelets were inhibited by the pretreatment with poly(RGD) in a concentration-dependent manner, but were not inhibited by treatment with poly(R, G, D) in which three amino acids were randomly arranged and no segment of the RGD sequence was therefore formed (Saiki *et al.*, 1989 *a*). On the other hand, natural killer (NK) cells and macrophages in certain blood cell populations appear to have an important role in the destruction of metastatic tumour cells. The activation of macrophages and NK cells by various immunostimulants led to the reduction of metastatic colonisation (Herberman, 1984; Hanna, 1985; Fidler *et al.*, 1981).

In this study, we examine the effect of polypeptides containing repetitive structure of the RGD sequence on the lung metastases of tumour cells, and study their biological characterisation in the metastatic cascade in order to gain insight into the mechanism and the action of our selected polypeptide.

### Materials and methods

### *Mice*

Specific pathogen-free mice of the inbred C57BL/6 and BALB/c strains, female, 7–10-week-old, were purchased from the Shizuoka Laboratory Animal Centre, Hamamatsu, Japan. Mice were maintained in the Laboratory of Animal Experiments, the Institute of Immunological Science, Hokkaido University, under laminar air-flow conditions.

*Cells*

Highly metastatic B16-BL6 melanoma cells, obtained by an *in vitro* selection procedure for invasion (Hart, 1979), were kindly provided by Dr I.J. Fidler (M.D. Anderson Cancer Center, Houston, Tx, USA). B16-BL6 melanoma and Lewis lung carcinoma (3LL) were derived from C57BL/6 mice and a colon 26 carcinoma was derived from BALB/c mice. These cells were maintained as monolayer cultures in Eagle's minimal essential medium (MEM) supplemented with 7.5% fetal bovine serum (FBS), vitamin solution, sodium pyruvate, non-essential amino acid and L-glutamine.

*Synthetic polypeptide analogues and other reagents*

The synthetic polypeptides used in this study and their abbreviations (based on the single-letter amino acid code) are given in Table I. Polypeptides with an Arg-Gly-Asp (RGD) sequence derived from fibronectin or the Tyr-Ile-Gly-Ser-Arg (YIGSR) sequence from laminin, as well as their related analogues, were prepared by the synthesis of the monomer peptide of the RGD or YIGSR sequences by the conventional method and subsequent polymerisation procedures with diphenylphosphoryl azide (DPPA), as described elsewhere (Nishi *et al.*, 1980, 1987). All the amino acids used in this study were of the L-form. Poly(RGD) or poly(YIGSR) consist of a sequential structure of the RGD or YIGSR sequences respectively, whereas poly(R, G, D) consists of a randomly arranged structure of three amino acids. Hence, in the sequence of poly(RGD), the G residue always lies between the R and D residues, and the -RGD- sequence is present as a segment. In the sequence of poly(R, G, D), on the other hand, three amino acids are randomly arranged without rule and the probability of their forming the -RGD- sequence is statistically very small. Copoly(RGD, K) and copoly(RGD, YIGSR) designate the polypeptides randomly arranged by RGD tripeptide and either K or YIGSR respectively, in which -RGD- and/or -YIGSR- sequences are always present as a segment.

Viscometric measurements and SDS-polyacrylamide gel electrophoresis showed that the polypeptides were approximately 10 kDa in average molecular weight. They were then dissolved in $Ca^{2+}$, $Mg^{2+}$-free phosphate-buffered saline (PBS). Purified mouse fibronectin was purchased from the Seikagaku Kogyo Co. Ltd (Tokyo, Japan). Arg-Gly-Asp-Ser (RGDS) was purchased from BACHEM Feinchemikauen AG (Switzerland). Pronase P was obtained from Kaken Chemicals Co. Ltd (Tokyo, Japan). All reagents and media used in this study were endotoxin free (approximately $< 1.0$ ng ml$^{-1}$) as determined by a colorimetric assay (Pyrodick, Seikagaku Kogyo Co., Tokyo, Japan).

*Microassay for cell adhesion*

The adhesion assay was carried out by the method described previously (Saiki *et al.*, 1986, 1989 *b*). B16-BL6 melanoma cells in an exponential growth phase were incubated for 24 h in MEM containing 5% FBS supplemented with 0.3 $\mu$Ci ml$^{-1}$ $^{125}$I-iododeoxyuridine ($^{125}$I-IUdR) (specific activity, 200 mCi mmol$^{-1}$, New England Nuclear, Boston, MA, USA). The cells were washed twice in warm PBS to remove unbound radiolabels, harvested by adding 0.02% EDTA in PBS for 1 min at 37°C and resuspended in cold serum-free MEM to form a single suspension of cells. $^{125}$I-IUdR-labelled tumour cells ($2 \times 10^4$) in a volume of 0.05 ml well$^{-1}$ were added to microculture wells pre-coated with synthetic polypeptides or fibronectin. The cultures were incubated at 37°C for 20 min unless otherwise stated and then washed four times with PBS to remove unattached cells. The remaining substrate-bound tumour cells were lysed with 0.1 ml of 0.1 N NaOH. The lysate was absorbed by cotton swabs and monitored for radioactivity by gamma counting. The binding capacity (no. of cells bound per substrate) was expressed as follows:

$$\text{Binding capacity} = \frac{\text{c.p.m. of targets bound to substrate}}{\text{c.p.m. of total tumour cells added}} \times \text{total number of tumour cells added}$$

*Experimental and spontaneous metastases assay*

Experimental metastasis was determined by means of tumour cell injection into the lateral tail veins of mice. Briefly, tumour cells ($5 \times 10^4$) were admixed with various concentrations of polypeptides in PBS and immediately 0.2 ml of these suspensions was injected into the lateral tail vein of syngeneic mice. The mice were killed 14 days after the inoculation of the tumour cells. In the spontaneous metastasis assay, mice were injected subcutaneously with B16-BL6 melanoma cells ($5 \times 10^5$) into the right hind footpad. Polypeptides were administered i.v. on various days after tumour inoculation, and the primary tumours were surgically removed by amputation on day 21. Mice were killed 14 days after the amputation. The lungs were fixed in Bouin's solution and the lung tumour colonies were counted under a dissecting microscope. The survival time of the animals given i.v. injections of tumour cells admixed with or without polypeptides was also determined by allowing the animals to live until they succumbed naturally from the tumour burden. Animals were autopsied at the time of death to verify the presence of the tumour in the lungs. The per cent survivors was calculated as a function of time.

*Platelet aggregation*

Blood was obtained from C57BL/6 mice by puncturing the retro-orbital plexus, using heparin (10 units ml$^{-1}$ in final concentration) as an anticoagulant. Platelet-rich plasma (PRP) was prepared by centrifugation at 160 *g* for 15 min at room temperature. PRP was adjusted to an appropriate concentration with platelet poor plasma (PPP), which had been prepared by centrifugation at 1,000 *g* for 10 min. Only plastic tubes and siliconised microcapillaries were used for these procedures. Platelet aggregation was measured in a dual aggregometer Model 440 (Chrono-Log, USA) at 37°C by constant stirring at 1,000 r.p.m. Variable concentrations of B16-BL6 tumour cells suspended in PBS were added to 0.25 ml of PRP which was then preincubated both with and without polypeptides for 5-7 min. The aggregometer was calibrated with PRP to express 100% optical transmission and with PPP to express 0% optical transmission.

*Organ distribution and retention of radiolabelled tumour cells*

B16-BL6 melanoma cells in the exponential growth phase were labelled with $^{125}$I-IUdR, as described above. $^{125}$I-IUdR-

**Table I** Synthetic polypeptides used in this study

| *Polypeptide* | *Single-letter abbreviation* |
|---|---|
| ---Arg-Gly-Asp-Arg-Gly-Asp-Arg-Gly-Asp-Arg-Gly-Asp-Arg--- | poly(RGD) |
| ---Arg-Gly-Asp-Lys-Lys-Arg-Gly-Asp-Arg-Gly-Asp-Lys-Arg--- | copoly(RGD, K) |
| ---Arg-Gly-Asp-Arg-Gly-Asp-Tyr-Ile-Gly-Ser-Arg-Arg-Gly-Asp--- | copoly(RGD, YIGSR) |
| ---Tyr-Ile-Gly-Ser-Arg-Tyr-Ile-Gly-Ser-Arg-Tyr-Ile-Gly-Ser--- | poly(YIGSR) |
| ---Arg-Asp-Arg-Gly-Asp-Gly-Asp-Asp-Arg-Gly-Gly-Arg-Asp-Gly--- | poly(R, G, D) |

labelled tumour cells ($2 \times 10^4$) in a volume of 0.2 ml were injected i.v. with or without polypeptide into the lateral tail vein of the C57BL/6 mice. Mice were exsanguinated at times ranging from 30 min to 24 h after the injection. The lungs, liver, spleen, kidneys and blood were collected from each mouse, and rinsed in 70% ethanol. The radioactivity in each organ was measured in a gamma counter.

*Labelling of synthetic polypeptide*

Copoly (RGD, K) was iodinated with Bolton-Hunter reagent according to the conventional procedure. Briefly, 2 mg copoly(RGD, K) was dissolved in 20 µl PBS and added to 1 mCi Bolton-Hunter reagent (*N*-succinimidyl 3-(4 hydroxy-3,5-$^{125}$I-diiodophenyl) propionate, specific activity 2,000 Ci mmol$^{-1}$, New England Nuclear, Boston, MA, USA) freshly dried from a solution in benzene. After agitation of the mixture at 4°C overnight, the reaction was quenched by the addition of 5 µl 1 M glycine in a borate buffer. Iodinated polypeptide was separated from the byproducts by gel filtration on a Sephadex G-25 which was equilibrated and eluted with a 0.05 M phosphate buffer (pH 7.5) containing 0.25% (w/v) gelatin. The $^{125}$I-labelled polypeptide thus obtained was confirmed by the absorbence at 280 nm in a spectrophotometer.

*Statistical analysis*

The statistical significance of differences between the groups was determined by applying Student's two-tailed *t* test unless otherwise mentioned.

### Results

*Inhibition of spontaneous and experimental lung metastases by polypeptides*

We first examined the effect of polypeptides containing the RGD and/or YIGSR sequence on lung metastasis of B16-BL6 melanoma in the spontaneous metastasis model (Table II). Multiple i.v. administrations of 100 µg poly(RGD), poly(YIGSR) or copoly(RGD, YIGSR) significantly reduced the number of lung tumour colonies ($P < 0.01$, 0.05 or 0.001 respectively). Copoly(RGD, YIGSR), in which the RGD and YIGSR sequences were randomly arranged at a 1:1 molar ratio, inhibited tumour metastasis more effectively than either poly(RGD) or poly(YIGSR). We also observed that polypeptides containing RGD sequence inhibited the experimental lung metastases after i.v. co-injection with different original metastatic tumour cells such as B16-BL6 melanoma, 3LL carcinoma and colon 26 carcinoma, but poly(R, G, D), which is a random homologue of poly(RGD), did not (Saiki *et al.*, 1989 *b*, c). The survival rate of mice given i.v. injections of B16-BL6 melanoma cells admixed with poly(RGD), poly(R, G, D) or RGD tripeptide was also determined (Figure 1). In this experiment, 50% of the mice which received untreated tumour cells succumbed to the tumour burden within 25 days of the injection. Similar survival rates were observed in the group of mice which received B16–BL6 cells admixed with poly(R, G, D) or RGD. The group that received tumour cells together with poly(RGD) showed a significantly enhanced survival rate ($P < 0.01$ by Mann–Whitney *U* probability test), but virtually all the mice had succumbed within 50 days of the i.v. injection of the tumour cells.

*Effect of anti-asialo GM1 serum, 2-chloroadenosine and carrageenan on poly(RGD)-mediated inhibition of tumour metastasis*

Since NK cells or macrophages in the circulation play an important role in the inhibition of tumour metastasis, we investigated whether or not poly(RGD) can stimulate NK cells or macrophages to induce the inhibition of tumour metastasis. Anti-asialo GM1 serum can selectively eliminate NK cells (Habu *et al.*, 1981) and 2-chloroadenosine (Saito & Yamaguchi, 1985), and carrageenan were macrophage toxic substances. Table III shows that the pretreatments with anti-asialo GM1 serum, 2-chloroadenosine or carrageenan enhanced the frequency of experimental metastasis as compared with the frequency found among untreated normal mice. The co-injection with poly(RGD) led to a significant reduction of lung tumour colonies in both untreated and treated mice.

*Effect of poly (RGD) on the interaction between tumour cells and platelets*

Several investigators have reported that platelets play an important role in the regulation of metastatic seeding. We therefore examined the effect of platelets and/or poly(RGD) on tumour cell adhesion to fibronectin-coated substrates. Figure 2 shows that B16-BL6 cells attached themselves to the fibronectin-coated substrate, but poly(RGD) led to a significant inhibition of tumour cell-adhesion to the substrate ($P < 0.001$). On the other hand, PRP dramatically enhanced the adhesion of B16-BL6 cells to the fibronectin-substrate in an assay on only 10 min, whereas PPP did not. The enhanced adhesion of tumour cells in the presence of PRP was dramatically inhibited by the addition of poly(RGD). These results indicate that poly(RGD) is able to inhibit the adhesion of tumour cells to immobilised fibronectin, as well as cell adhesion enhanced by platelets. We also investigated the effect of poly(RGD) on the platelet aggregation induced by tumour cells *in vitro*. Platelet aggregation elicited by B16-BL6 cells was monitored by a dual aggregometer (Figure 3). Poly(RGD) at a concentration of 100 µg ml$^{-1}$ inhibited the platelet aggregation induced by B16–BL6 cells. In contrast,

**Table II** Effect of polypeptides on spontaneous lung metastases by an intra-footpad injection of B16–BL6 melanoma

| *Administered i.v. with* | *Dose (µg per mouse)* | *No. of lung metastases on day 35 mean ± s.d. (range)* | $P^a$ |
|---|---|---|---|
| Untreated (PBS) | – | 66 ± 18 (34– 80) | |
| Poly(RGD) | 100 | 36 ± 8 (30– 46) | <0.01 |
| Poly(YIGSR) | 100 | 28 ± 24 (12– 64) | <0.05 |
| Copoly(RGD, YIGSR) | 100 | 12 ± 8 ( 2– 18) | <0.001 |
| RGD | 100 | 54 ± 22 (38– 90) | |
| YIGSR | 100 | 60 ± 38 (20–116) | |

Five C57BL/6 mice per group were administered i.v. with polypeptides on days 7, 9, 11, 13, 15, 17 and 19 after tumour inoculation. Primary tumours were surgically removed on day 21 and mice were sacrificed 2 weeks after tumour excision. [a]Compared with the control by Student's two-tailed *t* test.

**Figure 1** Effect of i.v. administration of polypeptides on the survival of C57BL/6 mice injected with B16-BL6 melanoma cells. Mice were injected i.v. with B16-BL6 cells ($5 \times 10^4$) together with medium (○—○), poly(RGD) (●—●), RGD (□—□) or poly (R, G, D) (■—■) and animal survival was monitored as a function of time.

**Table III** Effect of anti-asialo GM1 serum, 2-chloroadenosine or carrageenan on poly(RGD)-mediated inhibition of experimental tumour metastasis

| *Treatment of mice* | *Poly(RGD)* | *No. of lung metastases on day 14 mean ± s.d. (range)* | *P*[a] |
|---|---|---|---|
| None | – | 119 ± 16 (101–138) | |
| | + | 57 ± 7 ( 48– 65) | <0.001 |
| Anti-asialo GM1 20 µl i.v. | – | 144 ± 17 (124–164) | |
| | + | 59 ± 14 ( 44– 76) | <0.001 |
| 2-chloroadenosine 50 µg i.v. | – | 345 ± 28 (302–370) | |
| | + | 21 ± 5 ( 18– 27) | <0.001 |
| Carrageenan 1.2 mg i.p. | – | 332 ± 20 (304–352) | |
| | + | 39 ± 14 ( 25– 58) | <0.001 |

B16–BL6 melanoma cells (5 × $10^4$) were injected i.v. with or without 100 µg poly(RGD) into groups of control C57BL/6 mice or mice pretreated 24 h earlier with the indicated agents or antiserum. Lung tumour colonies were determined 14 days after tumour inoculation. [a]Compared with its respective untreated control (PBS) by Student's two-tailed *t* test.

**Figure 2** Effect of platelets and/or poly(RGD) on the adhesion of B16–BL6 cells to fibronectin-coated substrates. $^{125}$I-IUdR-(2 × $10^4$) were added to the wells coated with 5 µg ml$^{-1}$ fibronectin in the presence or absence of platelets (5 × $10^5$ µl$^{-1}$) and/or 100 µg ml$^{-1}$ poly(RGD), and incubated at 37°C for 10 min.

poly(R, G, D), in which three amino acids are randomly arranged, was not able to suppress the aggregation. Typical heterotypic cell aggregations between B16–BL6 cells and platelets, or the aggregation between platelets themselves, were microscopically observed after their co-incubation for 30 min (Saiki *et al.*, 1989 *a*). The cell aggregations were completely inhibited by the addition of 200 µg ml$^{-1}$ poly(RGD) but not by the addition of poly(R, G, D). These results clearly indicated that the adhesive interactions between tumour cells and platelets or between platelets on their own were completely inhibited by poly(RGD).

### *Organ localisation and retention of B16–BL6 melanoma cells co-injected with poly(RGD)*

To investigate the mechanisms responsible for a core sequence(RGD)-mediated inhibition of tumour colonisation, we tested the organ distribution and retention of $^{125}$I-IUdR-labelled tumour cells to see whether or not the co-injection of B16–BL6 melanoma cells with poly(RGD) can lead to a decrease in the arrest of tumour cells in the capillary bed of the chosen organ. Mice were killed at various times after the co-injection, and their visceral organs were collected and monitored for radioactivity in a gamma counter. The data of a representative experiment (one of three) are shown in Table IV. Significantly lower values were found in the lungs of mice at 4 and 24 h after the co-injection with poly(RGD). However, there are no discernible differences between control and poly(RGD) injected mice in the arrest and retention of labelled tumour cells in liver, spleen, kidneys and blood after tumour injection (data not shown).

### *Clearance of $^{125}$I-labelled polypeptide*

We recently found that the clearance of $^{125}$I-labelled poly(RGD, K) in the circulation was biphasic and rapid at an early phase after the i.v. injection (Saiki *et al.*, 1989 *b*). We next investigated the possibility that the polypeptide can be decomposed to by-products in the circulation. Figure 4 shows the separation patterns of the polypeptide by chromatography. Analysis of the column eluate revealed a main peak of radioactivity of untreated $^{125}$I-labelled poly(RGD, K) in the void volume. This main peak of $^{125}$I-labelled polypeptide was observed to decrease as a result of either the treatment with 75% fresh mouse serum or 1 mg ml$^{-1}$ pronase, and a new broad peak generated around the fraction number 27. In contrast, polypeptide treated with heat (56°C, 30 min) inactivated serum showed a similar

**Figure 3** Inhibition of tumour-induced platelet aggregation by polypeptides. Heparinised PRP ($5 \times 10^5\,\mu l^{-1}$) at a volume of 250 µl was treated with PBS (**a**), poly(R, G, D) (**b**, 100 µg ml$^{-1}$) and poly(RGD) (**c**, 100 µg ml$^{-1}$) 7 min before the addition of B16–BL6 melanoma cells (↑, $10^6$ ml$^{-1}$).

eluting pattern to untreated polypeptide. This result indicates that $^{125}$I-labelled polypeptide can be decomposed into small molecular weight fragments by a serum or pronase treatment *in vitro*.

### Discussion

Since the adhesive interactions between tumour cells and host cells or components of extracellular matrix play a crucial role in the progress of tumour metastases throughout a series of complex events, several attempts have been made to control the mechanisms involved in tumour cell adhesion during the metastatic cascade. In a previous report (Barsky *et al.*, 1984), the co-injection of tumour cells with purified laminin followed by i.v. injection enhanced pulmonary metastasis, whereas an enzymatic fragment of laminin inhibited the metastasis. Recently, synthetic oligopeptides containing characteristic sequences in cell-binding domains of adhesion molecules (GRGDS derived from fibronectin (Humphries *et al.*, 1986, 1988) or YIGSR derived from laminin (Iwamoto *et al.*, 1987)) have been shown to inhibit lung metastases when co-injected i.v. with metastatic tumour cells. We have synthesised some original polypeptides consisting of the repetitive structure of a common core sequence in cell adhesion molecules, such as poly(RGD) or poly(YIGSR), and found that these polypeptides are able to inhibit experimental lung metastases of B16–BL6 melanoma cells more effectively than such oligopeptides as RGD, RGDS or YIGSR on a weight basis (Saiki *et al.*, 1989 *c*). Furthermore, intratumoural or intravenous administrations of poly(RGD) into tumour-beaning mice (in a spontaneous metastasis model) resulted in a striking reduction of lung tumour colonies (Saiki *et al.*, 1989 *b*).

To extend our previous work on the inhibition of lung metastasis of tumour cells by a polypeptide containing RGD sequence, we here examine the inhibitory effect of the polypeptides on lung metastasis of tumour cells and their biological characterisation after the administration. Multiple i.v. administrations of poly(RGD), poly(YIGSR) or their hybrid polypeptide copoly (RGD, YIGSR) resulted in the reduction of lung tumour colonies (Table II), but did not affect the primary tumour size at the time of tumour excision (data not shown). The co-injection of B16–BL6 melanoma cells with poly(RGD) significantly enhanced the survival rate as compared with untreated control, poly(R, G, D) or RGD (Figure 1). These results indicate that derivatives of RGD- and/or YIGSR-containing polypeptides may be potentially useful in the prevention of cancer metastasis.

Once in circulation, metastatic tumour cells encounter various host cells or components (Fidler, 1984). Metastasising tumour cells, because of their adhesive properties, interact with host cells such as lymphocytes, NK cells and monocytes which are believed to be particularly important in killing these tumour cells, thus implying that the bloodstream may provide an inhospitable environment for the circulating tumour cells. In contrast, platelets have been reported to enhance the metastatic dissemination of tumour cells at several of the metastatic stages (Pearlstein *et al.*, 1980). The adhesive interaction between tumour cells and/or platelets may form homotypic or heterotypic cell clumps and aggregations which can subsequently be arrested and extravasated (Terranova *et al.*, 1982, 1984). Such interactions may also lead to an enhancement and stabilisation of tumour cell arrest in capillary vessels by increasing the size of tumour cell emboli as well as by shielding the tumour cells from immune response. As shown in Figure 3, tumour-induced platelet aggregation was completely inhibited by adding poly(RGD) but not by unrelated random polypeptide, poly(R, G, D). In addition, poly(RGD) also inhibited the adhesion of B16–BL6 cells to fibronectin-coated substrate (Figure 2). We have recently reported that the peptides or polypeptides containing the RGD sequence, when added freely in solution, are able to inhibit the tumour cell adhesion to fibronectin substrate, while unrelated peptides such as Arg-Gly-Glu-Ser (RGES) or His-Gly-Gly (HGG) are not able to do this (Saiki *et al.*, 1989 *b*). These results clearly demonstrate that the cell adhesion-inhibiting activities of poly(RGD) depend on a specific

**Table IV** Lung retention of $^{125}$I-IUdR-labelled B16–BL6 melanoma cells co-injected with poly(RGD) into C57BL/6 mice

| Treatment[a] | Radioactivity in lungs (c.p.m. ± s.d.)[b] | | |
|---|---|---|---|
| | *0.5 h* | *4 h* | *24 h* |
| PBS | 6528 ± 1604 (42.6%)[c] | 2034 ± 284 (13.2%) | 498 ± 51 (3.2%) |
| Poly(RGD) | 8723 ± 1850 (56.9%) | 1121 ± 281 ( 7.3%)[d] | 45 ± 19 (0.3%)[e] |

[a]$^{125}$I-IUdR-labelled B16–BL6 cells ($2 \times 10^4$ per mouse) were injected with or without 500 µg poly(RGD) into the lateral tail vein of C57BL/6 mice. At the indicated times, mice were killed and radioactive elements retained in the lung were measured. [b]Results are mean c.p.m. ± s.d. of three mice per group. [c]Parentheses represent % radioactivity of the input (15308 ± 1605 c.p.m. per $2 \times 10^4$ cells). [d]$P < 0.02$, [e]$P < 0.001$ as compared with untreated control (PBS) by Student's two-tailed *t* test.

**Figure 4** Gel chromatography of $^{125}$I-labelled poly (RGD, K) treated with mouse serum or pronase. $^{125}$I-labelled poly (RGD, K) (10 μg) was treated with MEM medium (●—●), 75% fresh serum in MEM (○—○) or 75% heat (56°C, 30 min)-inactivated serum in MEM (+—+) for 16 h, or with 1 mg ml$^{-1}$ pronase in MEM for 3 h (•–•), and then applied to a column of Sephadex G-25 eluted with 0.05 M phosphate buffer (pH 7.5) containing 0.25% (w/v) gelatin. Fractions of 70 μl were collected and analysed for radioactivity. Arrows represent the peaks of $V_0$ and $^{125}$I-IUdR (mol.wt 352) respectively.

mechanism mediated by the RGD core sequence. This also suggests that tumour cells possess RGD-directed receptors on their surface (Cheresh *et al.*, 1987; Wewer *et al.*, 1987). Tumour cell adhesion to fibronectin was enhanced remarkably in the presence of platelets (Figure 2). It has been reported that the mechanism by which platelets enhance tumour cell adhesion to the subendothelial matrix is mediated by surface contact between tumour cells and platelets, and depends on a platelet membrane component and cytoskeleton (Menter *et al.*, 1987). During the metastatic process, tumour-induced platelet aggregation may provide an additional means of adhesion through the interaction of platelets with adhesion proteins such as fibrinogen and fibronectin, as well as the opportunity to consolidate the adhesion through a thrombotic formation caused by platelet activation and the deposition of fibrin. Poly(RGD) completely inhibited the platelet-enhanced adhesion of B16–BL6 cells to the fibronectin substrate (Figure 2). RGD-containing peptides have been shown to inhibit the interaction of adhesive protein such as fibrinogen, fibronectin and the von Willebrand factor with a platelet membrane, presumably by means of the glycoprotein complex IIb/IIIa which serves as a receptor on platelets for such adhesive proteins (Haverstick *et al.*, 1985; Plow *et al.*,1987). A IIb/IIIa-like-glycoprotein identified on endothelial cells may also serve as a matrix receptor (Charo *et al.*, 1986; Fitzgerald *et al.*, 1985). Thus the inhibition of platelet aggregation and platelet-enhanced tumour cell adhesion to fibronectin substrate by poly(RGD) strongly implicates that the RGD sequence competitively blocks any interaction between tumour cells, tumour-stimulated platelets (presumably the IIb/IIIa complex) and such adhesive proteins as fibrinogen and fibronectin. Further studies will be needed to determine these points.

To investigate the effect of poly(RGD) on the arrest and lodgement of tumour cells in organs, we examined the organ retention of radiolabelled B16–BL6 cells. The co-injection of the tumour cells with poly(RGD) led significantly to a reduced arrest of tumour cells in the lung at 4 and 24 h after the injection (Table IV). The inhibition of lung colonisation by poly(RGD) may therefore depend on the decrease in the arrest and retention of tumour cells in the lung as a result of the inhibition of the adhesive interaction. We recently reported that radiolabelled polypeptide was biphasically cleared out from the circulation by the i.v. injection. The respective half-life of polypeptide at early and late phase was approximately 15 min and 6 h (Saiki *et al.*, 1989 *b*). These results indicate that this polypeptide may promote cell loss from the lung over 24 h after the co-injection with tumour cells.

Poly(RGD) significantly inhibited the experimental lung metastasis in mice pretreated with anti-asialo GM1 serum, 2-chloroadenosine or carrageenan as well as untreated mice (Table III). Since poly(RGD) was still active when the contributions of NK cells and macrophages were removed from our system, its inhibitory mechanism is likely to be unrelated to the stimulation and activation of these cells. The poly(RGD) used did not affect direct cytotoxicity against a variety of cell, nor did it affect their cell growth and the aggregation of serum proteins (unpublished data).

In conclusion, we here indicate that a unique RGD-containing polypeptide can dramatically inhibit lung metastases in experimental and spontaneous metastases models by means of its ability to interfere with the cellular adhesive process of metastases. The survival rate of mice receiving tumour cells admixed with poly(RGD) was virtually enhanced relative to control groups. Poly(RGD) inhibited the ability of platelets to enhance the adhesion of tumour cells, and also led to a decrease in the arrest and retention of tumour cells in the lung after co-injection with tumour cells. A polypeptide containing a core sequence derived from cell adhesion molecules may thus provide a promising approach for a therapy in the control of cancer metastases.

This work was supported in part by Grants-in-Aid for Cancer Research from the Japanese Ministry of Education, Science and Culture; from the Japanese Ministry of Health and Welfare for Comprehensive 10-Year Strategy for Cancer Control; and for Scientific Research and for Developmental Scientific Research (no.62870023) from the Japanese Ministry of Education, Science and Culture; and for Scientific Research from the Japanese Ministry of Education, Science and Culture; by the Yamanouchi Foundation for Research on Metabolic Disorders; and also by a Grant-in-Aid for Special Project Research from Hokkaido University, Japan. We thank Ms M. Sato for secretarial assistance.

### References

AKIYAMA, S.K. & YAMADA, K.M. (1985). Synthetic peptides competitively inhibit both direct binding to fibroblast and functional biological assays for the purified cell-binding domain of fibronectin. *J. Biol. Chem.*, **260**, 10402.

BARSKY, S.H., RAO, C.N., WILLIAMS. J.E. & LIOTTA, L.A. (1984). Laminin molecular domains which alter metastasis in a murine model. *J. Clin. Invest.*, **74**, 843.

CHARO, I.F., FITZGERALD, L.A., STEINER, B., RALL, S.C., BEKEART, L.S. & PHILIPS, D.R. (1986). Platelet glycoproteins IIb and IIIa: evidence for a family of immunologically and structurally related glycoproteins in mammalian cells. *Proc. Natl Acad. Sci. USA*, **83**, 8351.

CHERESH, D.A., PYTELA, R., PIERSCHBAHER, M.D., KLIER, F.G., ROUSLAHTI, E. & REISFELD, R.A. (1987). An arg-gly-asp-directed receptor on the surface of human melanoma cells exist in a divalent cation-dependent functional complex with the disaloganglioside GD2. *J. Cell. Biol.*, **105**, 1163.

FIDLER, I.J. (1984). The Ernst W. Bertner Memorial Award Lecture: The evolution of biological heterogeneity in metastatic neoplasms. In *Cancer Invasion and Metastases: Biologic and Therapeutic Aspects*, Nicolson, G.L. & Milas, L. (eds) p.5 Raven Press: New York.

FIDLER, I.J., SONE, S., FOGLER, W.E. & BARNES, Z.L. (1981). Eradication of spontaneous metastases and activation of alveolar macrophages by intravenous injection of liposomes containing muramyl dipeptide. *Proc. Natl Acad. Sci. USA*, **78**, 1680.

FITZGERALD, L.A., CHARO, I.F. & PHILLIPS, D.R. (1985). Human and bovine endothelial cells synthesize membrane proteins similar to human platelet glycoproteins IIb and IIIa. *J. Biol. Chem.*, **260**, 10893.

GASIC, G.J., GASIC, T.B., GALANTI, N., JOHNSON, T. & MURPHY, S. (1973). Platelet tumor interactions in mice. The role of platelets in the spread of malignant disease. *Int. J. Cancer*, **11**, 704.

HART, I.R. (1979). The selection and characterization of an invasive variant of the B16 melanoma. *Am. J. Pathol.*, **97**, 587.

HAVERSTICK, D.M., COWAN, J.F., YAMADA, K.M. & SANTORO, S.A. (1985). Inhibition of platelet adhesion to fibronectin, fibrinogen, and von Willebrand factor substrates by a synthetic tetrapeptide derived from the cell-binding domain of fibronectin. *Blood*, **66**, 946.

HAYMAN, E.G., PEIRSCHBAHER, M.D. & RUOSLAHTI, E. (1985). Detachment of cells from culture substrate by soluble fibronectin peptides. *J. Cell Biol.*, **100**, 1948.

HABU, S., FUKUI, H., SHIMAMURA, K. & 4 others (1981). *In vivo* effects of anti-asialo GM1. I. Redution of NK activity and enhancement of transplanted tumor growth in nude mice. *J. Immunol.*, **127**, 34.

HANNA, N. (1985). The role of natural killer cells in control of tumor growth and metastasis. *Biochim. Biophys. Acta*, **780**, 213.

HERBERMAN, R.B. (1984). Possible role of natural killer cells and other effector cells in immune surveillance against cancer. *J. Invest. Dermatol.*, **83**, 137.

HUMPHRIES, M.J., AKIYAMA, S.K., KOMORIYA, A., OLDEN, K. & YAMADA, K.M. (1986). Identification of an alternatively spliced site in human plasma fibronectin that mediates cell type-specific adhesion. *J. Cell Biol.*, **103**, 2637.

HUMPHRIES, M.J., YAMADA, K.M. & OLDEN, K. (1988). Investigation of the biological effects of anti-cell adhesive synthetic peptides that inhibit experimental metastasis of B16–F10 murine melanoma cells. *J. Clin. Invest.*, **81**, 782.

HUMPHRIES, M.J., OLDEN, K. & YAMADA, K.M. (1986). A synthetic peptide from fibronectin inhibits experimental metastasis of murine melanaoma cells. *Science*, **233**, 467.

IWAMOTO, Y., ROBEY, F.A., GRAF, J. & 4 others, (1987). YIGSR, a synthetic laminin pentapeptide, inhibits experimental metastasis formation. *Science*, **238**, 1132.

JAMIESON, G.A., BASTIDA, E. & ORDINAS, A. (1987). Interaction of platelets and tumor cells. In *Platelets in Biology and Pathology III*, MacIntyre, D.E. & Gordon, J.L. (eds) p.161. Elsevier Science Publishers: New York.

KOHGA, S., KINJO, M., TANAKA, K., OGAWA, H., ISHIHARA, M. & TANAKA, N. (1981). Effect of 5-(2-chlorobenzyl)-4,5,6,7-terahydrothieno [3,2-C] pyridine hydrochloride (Tichlopidine), a platelet aggregation inhibitor, on blood-borne metastasis. *Cancer Res.*, **41**, 4710.

KORNBLIHTT, A.R., UMEZAKA, K., VIBE-PEDERSAN, K. & BARALLE, F.E. (1985). Primary structure of human fibronectin: differential splicing may generate at least 10 polypeptides from a single gene. *EMBO J.*, **4**, 1755.

MCCARTHY, J.B. & FURCHT, L.T. (1984). Laminin and fibronectin promote the haptotactic migration of B16 mouse melanoma cells *in vitro*. *J. Cell Biol.*, **98**, 1474.

MENTER, D.G., SLOANE, B.F., STEINERT, B.M. & 5 others (1987). Platelet enhancement of tumor cell adhesion to subendothelial matrix: role of platelet cytoskeleton and platelet membrane. *J. Natl Cancer Inst.*, **79**, 1077.

MURATA, J., SAIKI, I., AZUMA, I. & NISHI, N. (1989). Inhibitory effect of a synthetic polypeptide, poly (Tyr-Ile-Gly-Ser-Arg), on the metastatic formation of malignant tumor cells. *Int. J. Biol. Macromol.*, **11**, 97.

MUSSONI, L., POGGI, A., DEGAESTANO, G. & DONATI, M.B. (1978). Effect of ditazole, an inhibitor of platelet aggregation on a metastasizing tumor in mice. *Br. J. Cancer*, **37**, 126.

NISHI, N., HAGIWARA, K. & TOKURA, S. (1987). Convenient preparation of poly-L-arginine by the direct polymerization of *N*-protected L-arginine derivatives with diphenylphosphoryl azide or diethylphosphorocyanidate. *Int. J. Peptide Protein Res.*, **30**, 275.

NISHI, N., NAKAJIMA, B., HASEBE, N. & NOGUCHI, J. (1980). Polymerization of amino acid or peptides with diphenylphosphoryl azide (DPPA). *Int. J. Biol. Macromol.*, **2**, 53.

PEARLSTEIN, E., SALK, P.L., YOGEESWARAN, G. & KARPATKIN, S. (1980). Correlation between spontaneous metastatic potential, platelet-aggregating activity of cell surface extracts, and cell surface sialylation in 10 metastatic-variant derivatives of a rat renal sarcoma cell line. *Proc. Natl Acad. Sci. USA*, **77**, 4336.

PIERSCHBACHER, M.D. & RUOSLAHTI, E. (1982). Cell attachment activity of fibronectin can be duplicated by small synthetic fragments of the molecule. *Nature*, **309**, 30.

PIERSCHBACHER, M.D. & RUOSLAHTI, E. (1984). Variants of the cell recognition site of fibronectin that retain attachment-promoting activity. *Proc, Natl Acad. Sci. USA*, **81**, 5986.

PLOW, E.P., PIERSCHBACHER, M.D., ROUSLAHTI, E., MARGUERIE, G. & GINSBERG, M.H. (1987). Arginyl-Glycyl-Aspartic acid sequences and fibrinogen binding to platelets. *Blood*, **70**, 110.

SAIKI, I., IIDA, J., AZUMA, I., NISHI, N. & MATSUNO, K. (1989 *a*). Biological activities of synthetic polypeptides containing a repetitive core sequence (Arg-Gly-Asp) of cell adhesion molecules. *Int. J. Biol. Macromol.*, **11**, 23.

SAIKI, I., IIDA, J., MURATA, J. & 5 others (1989 *b*). The inhibition of the metastases of murine malignant melanoma by means of synthetic polymeric peptides containing core sequence of cell adhesive molecules. *Cancer Res.*, **49**, 3815.

SAIKI, I., MURATA, J., IIDA, J., NISHI, N., SUGIMURA, K. & AZUMA, I. (1989 *c*). The inhibition of murine lung metastasis by synthetic polypeptides [poly (arg-gly-asp) and poly (tyr-ile-gly-ser-arg)] with a core sequence of cell adhesion molecules. *Br. J. Cancer*, **59**, 194.

SAIKI, I., NAYAR, R., BUCANA, C. & FIDLER, I.J. (1986). A microassay for the rapid and selective binding of cells from solid tumors to mouse macrophages. *Cancer Immunol. Immunother.*, **22**, 125.

SAITO, T. & YAMAGUCHI, J. (1985). 2-Chloroadenosine: a selective lethal effect to mouse macrophages and its mechanism. *J. Immunol.*, **134**, 1815.

SASAKI, M., KATO, S., KOHNO, K., MARTIN, G.R. & YAMADA, Y. (1987). Sequence of the cDNA encoding the laminin B1 chain reveals a multidomain protein containing cystein rich repeats. *Proc. Natl Acad. Sci. USA*, **84**, 935.

SASAKI, M. & YAMADA, Y. (1987). The laminin B2 chain has a multidomain structure homologous to the B1 chain. *J. Biol. Chem.*, **262**, 17111.

SUZUKI, S., OLDBERG, A., HAYMAN, E.G., PIERSCHBACHER, M.D. & ROUSLAHTI, E. (1985). Complete amino acid sequence of human vitronectin deduced from cDNA. Similarity of cell attachment sites in vitronectin and fibronectin. *EMBO J.*, **4**, 2519.

TERRANOVA, V.P., LIOTTA, L.A., RUSSO, R.G. & MARTIN, G.R. (1982). Role of laminin in the attachment and metastasis of murine tumor cells. *Cancer Res.*, **42**, 2265.

TERRANOVA, V.P., WILLIAMS, J.E., LIOTTA, L.A. & MARTIN, G.R. (1984). Modulation of the metastatic activity of melanoma cells by lamanin and fibronectin. *Science*, **226**, 982.

TSURUO, T., IIDA, H., MAKISHIMA, M. & 4 others (1985). Inhibition of spontaneous and experimental tumor metastasis by the calcium antagonist verapamil. *Cancer Chemother. Pharmacol.*, **14**, 30.

WEWER, U.M., TARABOLETTI, G., SOBEL, M.E., ALBRECHTSEN, R. & LIOTTA, L.A. (1987). Role of laminin receptor in tumor cell migration. *Cancer Res.*, **47**, 5681.

YAMADA, K.M. & KENEDY, D.W. (1984). Dualistic nature of adhesive protein function: fibronectin and its biologically active peptide fragments can autoinhibit fibronectin function. *J. Cell Biol.*, **99**, 29.

YAMADA, K.M. & KENNEDY, K.W. (1987). Peptide inhibitors of fibronectin, laminin and other adhesion molecules: unique and shared features. *J. Cell. Physiol.*, **130**, 21.